# Physiological and Comparative Transcriptome Analysis Reveals the Mechanism by Which Exogenous 24-Epibrassinolide Application Enhances Drought Resistance in Potato (*Solanum tuberosum* L.)

**DOI:** 10.3390/antiox11091701

**Published:** 2022-08-30

**Authors:** Hao Zheng, Jie Ma, Wenli Huang, Hongmei Di, Xue Xia, Wei Ma, Jun Ma, Jiao Yang, Xiaomei Li, Huashan Lian, Zhi Huang, Yi Tang, Yangxia Zheng, Huanxiu Li, Fen Zhang, Bo Sun

**Affiliations:** 1 College of Horticulture, Sichuan Agricultural University, Chengdu 611130, China; 2 Bijie lnstitution of Agricultural Science, Bijie 551700, China; 3 Rice and Sorghum Research Institue, Sichuan Academy of Agricultural Sciences, Deyang 618000, China; 4 Vegetable Germplasm Innovation and Variety Improvement Key Laboratory of Sichuan, Chengdu 610300, China; 5 School of Agriculture and Horticulture, Chengdu Agricultural College, Chengdu 611130, China

**Keywords:** brassinosteroids, drought stress, photosynthesis, antioxidant system, abscisic acid, drought-responsive gene, potato

## Abstract

Drought stress is a key factor limiting the growth and tuber yield of potatoes (*Solanum tuberosum* L.). Brassinosteroids (BRs) have been shown to alleviate drought stress in several plant species; however, little is known about the physiological and molecular mechanisms by which BRs enhance drought resistance in potatoes. Here, we characterized changes in the physiology and transcriptome of the tetraploid potato variety ‘Xuanshu-2′ in response to drought stress after 24-epibrassinolide (EBR) pretreatment. The abscisic acid (ABA) content, photosynthetic capacity, and the activities of antioxidant enzymes were increased; the intercellular CO_2_ concentration, relative conductivity, reactive oxygen species, malondialdehyde, proline, and soluble sugar content were decreased after EBR pretreatment compared with plants under drought stress. Transcriptome analysis revealed 1330 differently expressed genes (DEGs) involved in the response to drought stress after EBR pretreatment. DEGs were enriched in plant hormone signal transduction, starch and sucrose metabolism, circadian rhythm, flavonoid biosynthesis, and carotenoid biosynthesis. DEGs associated with the BR signaling and biosynthesis pathways, as well as ABA metabolic pathways were identified. Our findings provide new insights into the mechanisms by which BRs enhance the drought resistance of potatoes.

## 1. Introduction

Potatoes (*Solanum tuberosum* L.) are the world’s most important tuber crop, and they are rich in starch, protein, amino acids, and vitamins [1,2]. Although they grow in arid and semiarid regions with prolonged periods without precipitation, potatoes are sensitive to water deficits [3], and lack of water can reduce plant performance and result in the loss of tuber yield [4]. Drought stress is one of the predominant factors affecting potato yield and quality. Therefore, exploring the physiological and molecular mechanisms in potatoes under drought stress and improving the drought resistance are effective strategies for enhancing the production and cultivation of potatoes.

Plant systems generally adapt to moderate drought stress and maintain physiological water balance by increasing root water uptake [5], closing the stomata to reduce water loss, and adjusting osmotic processes within tissues [4,5]. Some of the pathways activated in response to drought stress include phytohormone signaling pathways [6], as well as the production and mobilization of antioxidants and metabolites [6]. Drought stress leads to a reduction in the hydration of the membranes and proteins, and the concomitant excessive accumulation of reactive oxygen species (ROS) [7]. Excessive ROS triggers the accumulation of malondialdehyde (MDA), and induces damage to chloroplasts and mitochondria, and degrades the structure of cells [8,9]. Plants have evolved ROS scavenging mechanisms to mitigate and prevent damage caused by ROS, including enzymatic systems, such as superoxide dismutase (SOD), peroxidase (POD), catalase (CAT), and ascorbate peroxidase (APX), as well as nonenzymatic systems, such as ascorbic acid (AsA), glutathione (GSH), flavonoids, carotenoids, and proline [10]. Abscisic acid (ABA), which is a well-known “stress hormone”, is a key molecule involved in root-to-shoot signaling in response to drought stress [4] and a regulator of stomatal conductance [6]. In most instances, ABA controls stomatal aperture and the expression of stress-responsive genes [4], thereby reducing CO_2_ in the leaf, which limits photosynthesis and enhances tolerance to unfavorable conditions [6,11].

Brassinosteroids (BRs) are a group of polyhydroxylated plant steroid hormones that play a vital role in plant growth and development [12,13], as well as responses to different types of abiotic stresses, such as extreme temperatures, salinity, and drought [14,15,16,17]. Previous studies have shown that the application of exogenous BRs can improve the drought resistance of some crops, such as soybean [18], wheat [19,20], Indian mustard [21], tomato [22], maize [23,24], barley [25,26], peach [27], and lulo [28]. These studies of exogenous BR-mediated drought resistance have shown that exogenously applied BRs can (1) enhance photosynthetic performance [18,19,23,24,28]; (2) activate the antioxidant system [22,23,24,25]; (3) regulate phytohormone metabolism pathways and increase the endogenous ABA concentration [15,22]; and (4) regulate the expression of genes involved in drought-stress tolerance and ameliorate drought stress [20,24,27].

Endogenous BR signaling is also involved in the drought response and the coordination of plant growth and drought responses [12,29,30]. Plants typically respond to endogenous or exogenous BRs through BR signal transduction [31,32], and the BR signaling pathway involves a series of receptors, kinases, and regulators [14]. In *Arabidopsis*, overexpression of the BRI1-LIKE receptor homolog gene *BRL3* can confer drought stress tolerance [33], and the phosphorylates of RD26 BRASSINOSTEROID-INSENSITIVE 2 (BIN2) can promote drought signaling [34]. Conversely, the transcription factors WRKY46/54/70 interact with BES1 to inhibit the expression of drought-responsive genes, and play a positive role in BR-regulated growth, and play a negative role in drought responses [35]. Furthermore, BR signaling negatively regulates the activity of the AP2/ERF transcription factor TINY through BIN2 phosphorylation [29]; TINY positively regulates drought responses and inhibits BR-mediated growth through antagonistic interactions with TINY-BES1 in *Arabidopsis* [29]. In addition, the BR biosynthetic rate-limiting enzyme gene *AtCPD* [36], the BES/BZR transcription factor *TaBZR2* [20], and the BR-signaling kinase *ZmBSK1* [37] can enhance the drought resistance of potatoes, wheat, and maize, respectively. BRs generally regulate stress responses through complex molecular networks rather than via a linear pathway [23,30], and the crosstalk between BRs and ABA also adds additional complexity to the BR-mediated drought regulation [30].

Previous research examining the application of BRs in potatoes has mainly focused on tuberization and tuber yields [38,39], tuber sprouting [40,41], and root growth and salinity stress [42,43]. However, few studies have examined the application of exogenous BRs in potatoes under drought stress, and the physiological and molecular mechanisms by which BRs alleviate drought stress in potatoes remain unclear. Therefore, additional studies are needed to determine how BRs mediate the response to drought stress at the molecular, biochemical, and physiological levels [3]. Here, the physiological and molecular mechanisms by which a BR modulates the response of potatoes to drought stress were investigated. Exogenous application of 0.5 μmol L^−1^ of 24-epibrassinolide (EBR) was found to effectively alleviate drought stress in potatoes. We examined changes in plant growth attributes, photosynthetic capacity, the antioxidant system, and osmotic solutes of potatoes after four treatments: control (distilled water plus optimal growth conditions, WC), EBR plus optimal growth conditions (BC), distilled water plus drought (WD), and EBR plus drought (BD). Gene expression patterns of the four treatments were characterized by RNA-sequencing (RNA-seq). The results of our study provide new insights into the mechanisms underlying the BR-mediated drought responses of potatoes.

## 2. Materials and Methods

### 2.1. Plant Materials and Growth Conditions

Xuanshu-2, a moderately drought-tolerant tetraploid potato (*Solanum tuberosum* L.) cultivar, was used in the experiments. A pre-experiment was conducted using ‘Xuanshu-2′ potato plants at the rosette stage to determine the effective dose of EBR. A total of 180 plants were assigned to six groups and sprayed with 0 (control), 0.125, 0.25, 0.5, 1, and 2 μmol L^−1^ of EBR. Potato plants treated with 0.5 μmol L^−1^ of EBR had significantly ameliorated drought symptoms compared with other treatment groups and the control (Figure S1).

A total of 240 plants were selected and assigned to four groups (3 batches in each group, 20 plants in each batch). The treatments were as follows: (1) plants were pretreated with ddH_2_O for 3 d, and followed by 9 d of adequate watering (WC); (2) plants were pretreated with an equal volume of 0.5 μmol L^−1^ of EBR for 3 d, followed by 9 d of adequate watering (BC); (3) plants were pretreated with ddH_2_O for 3 d, followed by 9 d of drought (WD); (4) plants were pretreated with 0.5 μmol L^−1^ of EBR for 3 d, followed by 9 d of drought (BD). The day water began to be withheld was designated as 0 d. Samples were taken before EBR treatment (−3 d) and at 3-d intervals during drought treatment for measurements.

### 2.2. Water Content and ABA Content

The leaves of potato plants were removed and weighed immediately. The leaves were then oven-dried to a constant weight at 80 °C, and the dry weight was determined. The water content was calculated according to the following formula: (WF − WD)/WF × 100%. The ABA content was detected using the ABA ELISA kit (Qisong Biotechnology, Beijing, China).

### 2.3. Photosynthesis and Chlorophyll Fluorescence Measurements

*Pn*, Tr, Ci, and Gs of fully developed source leaves on branches (3rd to 5th from bottom) were measured using the LI-COR 6800 Photosynthesis System (Li-Cor, Lincoln, NE, USA). All measurements were conducted between 4 and 6 h after dawn (10:00 to 12:00 a.m.) under controlled conditions (27 °C, 400 µmol mol^−1^ OF CO_2_, 60% relative humidity). After dark adaptation for 30 min, the same leaves used for photosynthesis measurements were used to determine Fv/Fm and qN with the LI-COR 6800 Photosynthesis System (Li-Cor, Lincoln, NE, USA).

### 2.4. Content of Photosynthetic Pigments Content

The samples (0.1 g) were ground, extracted with 10 mL of ethanol, and centrifuged at 4000× *g* at room temperature for 5 min. The supernatant was collected and analyzed using the UV-1800 spectrophotometer (MAPADA, Shanghai, China); the absorbance was detected at 665 nm, 649 nm, and 451 nm to measure the content of chlorophyll and carotenoids. The content of pigments was expressed as mg g^−1^ fresh weight [44].

### 2.5. NBT and DAB Staining

The nitrotetrazolium blue chloride (NBT) staining method was based on the procedure described in [45]. Leaves were incubated in NBT staining solution (0.1% NBT, 10 mM potassium phos-phate buffer, pH 7.6) in the dark for 6 h; they were then boiled in 96% ethanol for 10 min before the blue precipitates were visualized. Histochemical detection of H_2_O_2_ was performed using the 3,3′-diaminobenzidine (DAB) staining method [45]. Leaves were incubated in DAB staining solution (1 mg mL-1 DAB, pH 3.8) in the dark for 8 h and decolorized as NBT. The intensity of brown coloration indicated the H_2_O_2_ content.

### 2.6. Quantification of MDA and Activities of SOD, POD, and CAT

MDA was measured according to the procedure described by [45]. Leaf tissue (0.5 g) was ground with 6 mL of 10% trichloroacetic acid, and centrifuged at 8000× *g* for 5 min; the supernatant (2 mL) was mixed with 2 mL of 0.6% thiobarbituric acid (TBA). The mixture was then boiled in water for 15 min and quickly cooled for centrifugation; the absorbance was then measured at 532 nm, 600 nm, and 450 nm, and the MDA content (μmol kg^−1^ fresh weight) was calculated.

To prepare the crude enzyme extract, leaf powder (0.5 g) was homogenized in a cooled extraction buffer containing 10 mL of 0.05 mol L^−1^ phosphate buffer (pH 7.8); after mixing the leaf powder suspension, it was centrifuged at 9000× *g* and 4 °C for 10 min, and the supernatant was collected for subsequent tests. Activities of SOD, POD, and CAT were determined following the procedures described by [45]. The activity of enzymes was presented as U g protein.

### 2.7. Relative Conductivity

Fresh complete leaves were soaked in a tube of 20 mL of ddH_2_O, and measurements were taken after 3 h at room temperature. Measurements of the solution were then taken again after boiling for 1 h. Distilled water without leaves was used as the control. Relative conductivity was calculated using the following formula: (%) = (Conductivity Initial sample-Conductivity Initial water/Conductivity Final sample-Conductivity Final water) × 100%.

### 2.8. Proline, Soluble Protein, and Soluble Sugar Content

To determine the content of proline, fresh leaves (0.5 g) were homogenized in 5 mL of 3% sulfosalicylic acid for 10 min at 90 °C and then cooled for centrifugation. The supernatant (2 mL) was treated with 2 mL of glacial acetic acid and 3 mL of acid ninhydrin, followed by 4 mL of toluene. The absorbance of the colored solutions was read on the UV-1800 spectrophotometer (MAPADA, China) at 520 nm [46].

To determine the content of soluble protein, leaf powder (0.5 g) was soaked in 10 mL of ddH_2_O and centrifuged for 5 min at 4000× *g*; then, 1 mL of the supernatant was transferred to a polypropylene tube. Coomassie brilliant blue G250 was added to the tube along with the supernatant. The absorbance was measured with the UV-1800 spectrophotometer (MAPADA, China) at 595 nm within 20 min after the reaction [44].

To determine the content of soluble sugars, leaf powder (0.5 g) was extracted in 10 mL of ddH_2_O for 20 min at 90 °C and centrifuged at 4000× *g* for 5 min. A combination of 1 mL of sample extract, 0.5 mL of anthrone-ethylacetate reagent, and 5 mL of concentrated sulfuric acid was homogenized and boiled for 5 min, and then rapidly cooled. The absorbance of the reaction mixtures was measured at 630 nm using the UV-1800 spectrophotometer (MAPADA, China) [44].

### 2.9. RNA Sequencing and Analysis of Differentially Expressed Genes

The leaves of WC, BC, WD, and BD plants 3 d after drought treatment were collected, and RNA-seq was performed using the Illumina HiSeqX10 platform by Annoroad Gene Technology Co., Ltd. (Beijing, China). Three biological replicates were performed for each of the four sample groups. After sequencing, the high-quality clean reads were obtained by removing reads containing adapters and high-quality sequences. High-quality sequences were then aligned to the reference genome using HISAT2 software. Analysis of gene expression levels was conducted based on fragments per kilobase per million fragments mapped (FPKM). Differential expression analysis of the two treatments was performed using the DESeq2 package, and differential expression genes (DEGs) were defined using the following criteria: |log2 Fold change| ≥ 1 and *p*-value < 0.05.

### 2.10. GO and KEGG Enrichment

Gene ontology (GO) enrichment analysis of DEGs was performed using the AmiGO 2 database (http://amigo.geneontology.org/amigo/, accessed on 2 December 2018). Kyoto encyclopedia of genes and genomes (KEGG) pathway enrichment analysis of DEGs was performed using the KEGG database (https://www.kegg.jp/kegg/, accessed on 2 December 2018). GO and KEGG terms with corrected *p*-value < 0.05 were considered significantly enriched.

### 2.11. Quantitative Real-Time PCR (qRT-PCR) Validation

RT-qPCR was performed following the instructions of the TB Green Premix Ex Taq II (Tli RNaseH Plus) kit on a Bio-Rad iCycler thermocycler (Bio-Rad, Hercules, CA, USA). Relative gene expression levels were normalized by comparison with the expression of potato elongation factor 1-α (*ef1α*) [47], and calculated using the formula 2^−ΔΔCT^ [48]. There were three independent biological repetitions for all qRT-PCR reactions, which were each run in three parallel reactions. The primers used in this study are listed in Table S1.

### 2.12. Statistical Analysis

Data were analyzed by two-way analysis of variance (ANOVA) using DPS 15.10. Differences among treatments were determined using least significant difference (LSD) tests, and the threshold for statistical significance was *p* < 0.05 (the significance of physiological parameters is shown in Table S2). Data were presented as the mean ± SD of three biological replicates. A heatmap was generated using TBtools software with a log scale [49]. A time-related trajectory analysis based on a principal component analysis (PCA) map was used to visualize temporal changes in drought stress between different treatments. A PCA was performed using SIMCA 13.0 software with unit variance scaling to determine the relationships among samples.

## 3. Results

### 3.1. Exogenous Application of EBR Delayed Plant Water Loss and Increased the ABA Content under Drought

**Figure 1:**
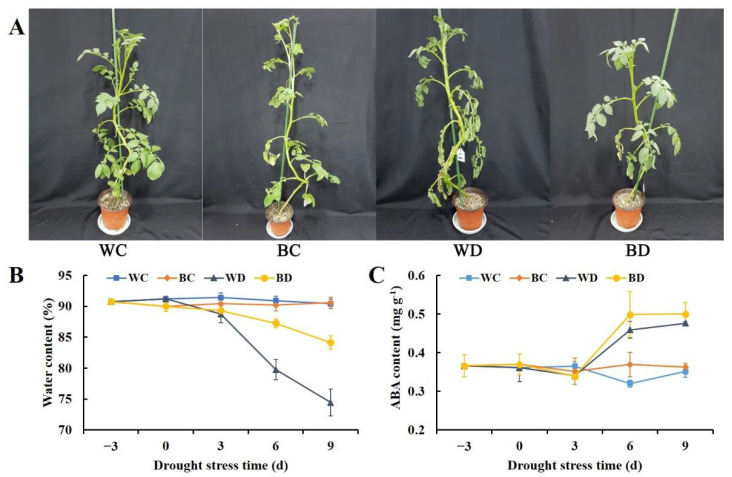
Effects of EBR pretreatment on the phenotype (**A**), water content (**B**), and ABA content (**C**) of potato plants exposed to drought. The plant phenotype is 9 d after drought treatment. WC, distilled water plus optimal growth conditions; BC, EBR plus optimal growth conditions; WD, distilled water plus drought; BD, EBR plus drought. Each data point represents the mean of three replicate samples, and vertical bars represent the standard deviation of the mean. The threshold for statistical significance was *p* < 0.05 using least significant difference (LSD) tests (the significance of physiological parameters is shown in Table S2) (below).

After 9 d of the experiment, plant phenotypes differed among the four treatments (Figure 1A). Normally watered plants grow well (WC and BC). The WD plants were severely wilted, and the middle and lower leaves had symptoms of curling. The BD plants showed less severe symptoms of drought stress, as only the lower leaves were slightly dehydrated and wilted. All physiological parameters and their corresponding significance in potato leaves with EBR pretreatment under drought are shown in Table S2. The EBR application alleviated water loss and increased the ABA content of potato leaves under drought (Figure 1B,C). The water content of the WD plants was significantly decreased compared with that of the WC plants at 3 d of drought stress; however, a decrease in the water content of the BD plants was not observed until 6 d. The water content was 1.1-fold higher in the BD plants than that of the WD plants at 6 d and 9 d. After 3 d of drought, ABA accumulated rapidly in the WD and BD plants, and the ABA content was 1.4-fold higher in the WD and the BD plants than in the WC and the BC plants at 6 d. The ABA content was higher in the BD plants than in the WD plants throughout the drought stress period, and it was 8% higher in the BD plants than in the WD plants at 6 d of drought. This indicates that drought stress increased the ABA content, and that the EBR pretreatment increased the ABA content more than drought stress alone.

### 3.2. Exogenous Application of EBR Enhanced the Photosynthetic Capacity under Drought

**Figure 2:**
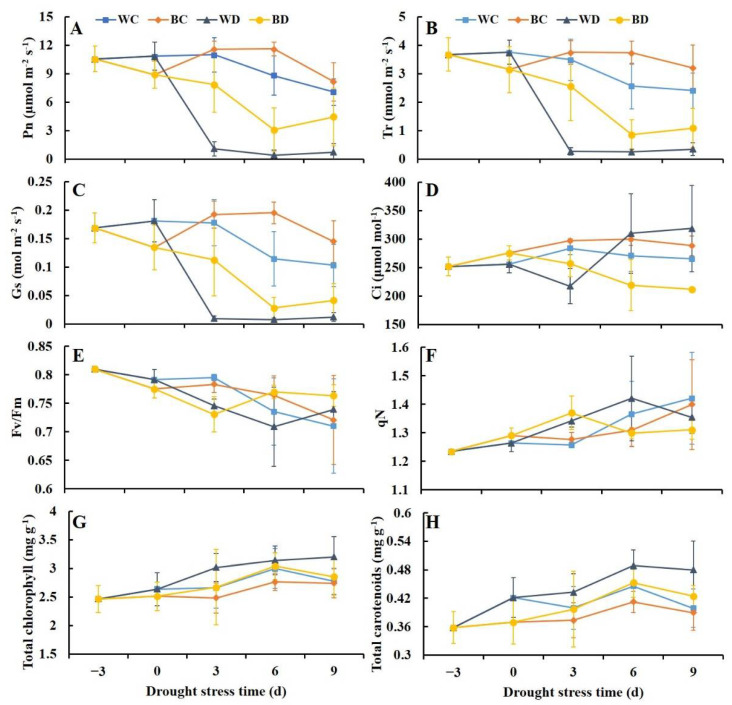
Effects of EBR pretreatment on photosynthetic parameters of potato plants exposed to drought. (**A**) Net photosynthetic rate (*Pn*); (**B**) transpiration rate (Tr); (**C**) stomatal conductance (Gs); (**D**) intercellular CO_2_ concentration (Ci); (**E**) maximum photochemical efficiency (*Fv*/*Fm*); (**F**) nonphotochemical quenching (qN); (**G**) chlorophyll content; (**H**) carotenoids content.

As the drought progressed, the net photosynthetic rate (*Pn*) decreased, and the decrease was especially sharp at 3 d of drought; *Pn* was near zero in the later part of the experimental period (Figure 2A). Changes in the transpiration rate (Tr) and stomatal conductance (Gs) were consistent with changes in *Pn* (Figure 2B,C). At 3 d of drought, *Pn*, *Tr*, and *Gs* decreased more slowly in the BD plants than in the WD plants, and *Pn*, *Tr*, and *Gs* were 7.3-, 9.4-, and 12.3-fold higher in the BD plants than in the WD plants, respectively. *Pn* was 6.2-fold higher in the BD plants than in the WD plants at 9 d of drought, and this difference was significant. Changes in the intercellular CO_2_ concentration (Ci) differed from changes in the other three gas exchange parameters (Figure 2D). In the WD plants, Ci first rapidly declined and then increased; by contrast, in the BD plants, Ci declined continuously, and it was only 71% and 66% in the WD plants at 6 d and 9 d, respectively.

Under drought stress, although the maximum photochemical efficiency (*Fv*/*Fm*) and nonphotochemical quenching (*qN*) values showed a slight decreasing and increasing trend, respectively, there were no significant differences between the WD and the BD plants (except for the *qN* value at 6 d of drought stress) (Figure 2E,F). In addition, the content of total chlorophyll and total carotenoids had no significant differences between the WD and the BD plants (Figure 2G,H).

### 3.3. Exogenous Application of EBR Enhanced Antioxidant Enzyme Activity and the Scavenging of ROS under Drought

**Figure 3:**
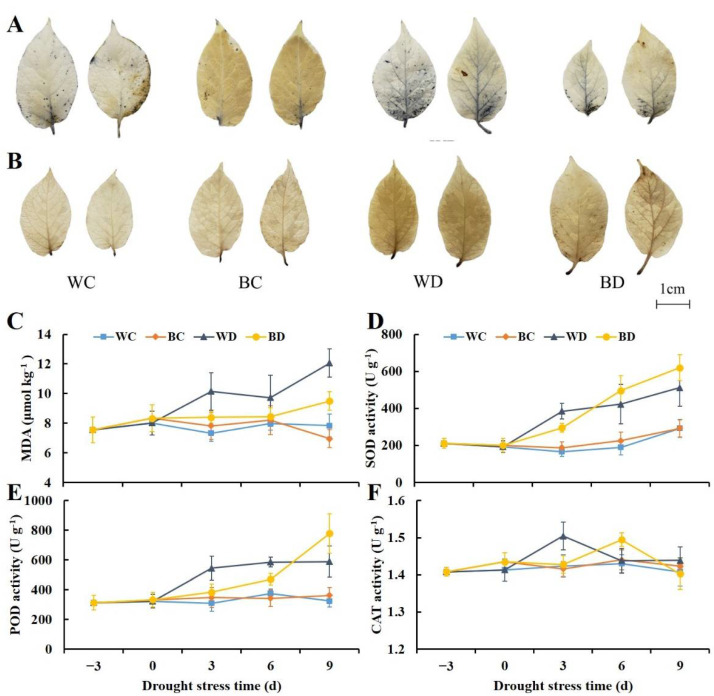
Effects of EBR pretreatment on the accumulation and scavenging of ROS in potato plants exposed to drought. (**A**) Histochemical detection of O^2−^ with nitrotetrazolium blue chloride (NBT) staining in potato leaves 9 days after drought treatment; (**B**) histochemical detection of H_2_O_2_ with 3,3-diaminobenzidine (DAB) staining in potato leaves 9 days after drought treatment; (**C**) MDA content; (**D**) SOD activity; (**E**) POD activity; (**F**) CAT activity.

Nitrotetrazolium blue chloride (NBT) and 3,3-diaminobenzidine (DAB) staining confirmed the accumulation of O^2−^ and H_2_O_2_ in drought stressed leaves, and there was a marked difference between the treatments with and without EBR pretreatment (Figure 3A,B). The main veins, lateral veins, and part of the reticulate veins of the WD leaves were stained dark blue by the NBT, and the leaf surfaces were stained brown by the DAB. Conversely, changes in the color of EBR-treated leaves were not obvious, indicating that EBR treatment considerably reduced O^2−^ and H_2_O_2_ accumulation in leaves under drought.

The MDA content in potato plants increased continuously under drought, and it was 1.5-fold higher in the WD plants than in the WC plants at 9 d of drought stress. The MDA content was lower in the BD plants than in the WD plants, and it remained low throughout the drought treatment (Figure 3C). The activities of SOD and POD increased consistently under drought stress, and the rate of increase in the WD plants was fastest in the early stages of drought stress. The highest activities of both enzymes were observed at 3 d of drought stress in WD, 2.3- and 1.3-fold higher in the WD plants than in the WC plants and 1.8- and 1.4-fold higher in the WD plants than in the BD plants, respectively. A rapid increase in the BD plants was observed in the later part of the experimental period, and the activities of SOD and POD were 1.2- and 1.3-fold higher in the BD plants than in the WD plants at 9 d, respectively (Figure 3D,E). Changes in the CAT activity differed from changes in the SOD and POD activities; fluctuations in the CAT activity were small in the four treatments, and the CAT activity generally ranged from 1.4 to 1.5 U g^−1^. The peak of the CAT activity was delayed in the BD plants compared with the WD plants (Figure 3F).

### 3.4. Exogenous Application of EBR Alleviated Increases in Relative Conductivity and Osmotic Solutes under Drought

**Figure 4:**
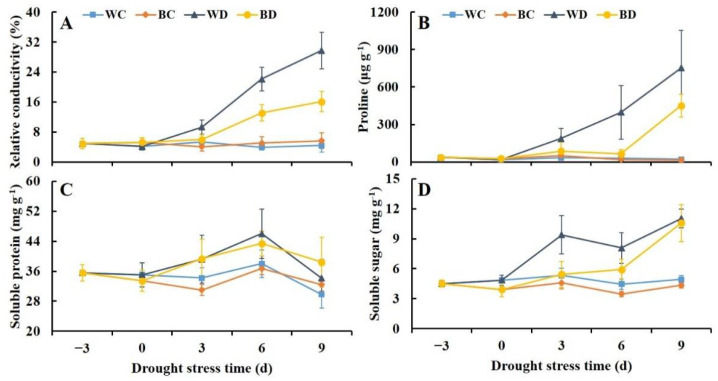
Effects of EBR pretreatment on relative conductivity (**A**), proline (**B**), soluble protein (**C**), and soluble sugar (**D**) of potato plants exposed to drought.

The relative conductivity in the WD and the BD plants increased continuously throughout the experimental period, and the relative conductivity was 6.8-fold higher in the WD plants than in the WC plants; the relative conductivity of the BD plants was only 54% of that of the WD plants at 9 d of drought stress (Figure 4A). The proline content increased under drought stress, and it was 5.6- to 36.3-fold higher in the WD plants than in the WC plants at 9 d of drought stress. The proline content was relatively stable in the BD plants in the early stages of drought stress (3 d), and no differences in the proline content were observed between the BD plants and the WC plants during this period. The proline content increased rapidly after 6 d of drought stress, and the proline content of the BD plants was 60% of that of the WD plants at 6 d (Figure 4B). Drought stress caused significant increases in the content of soluble sugar, and in the WD plants it increased sharply at 3 d and continued to increase; however, an increase in the content of soluble sugar was not observed in the BD plants until 6 d. In addition, the content of soluble protein had no significant differences between the WD and the BD plants (Figure 4C,D).

### 3.5. Time-Related Trajectory Analysis

**Figure 5:**
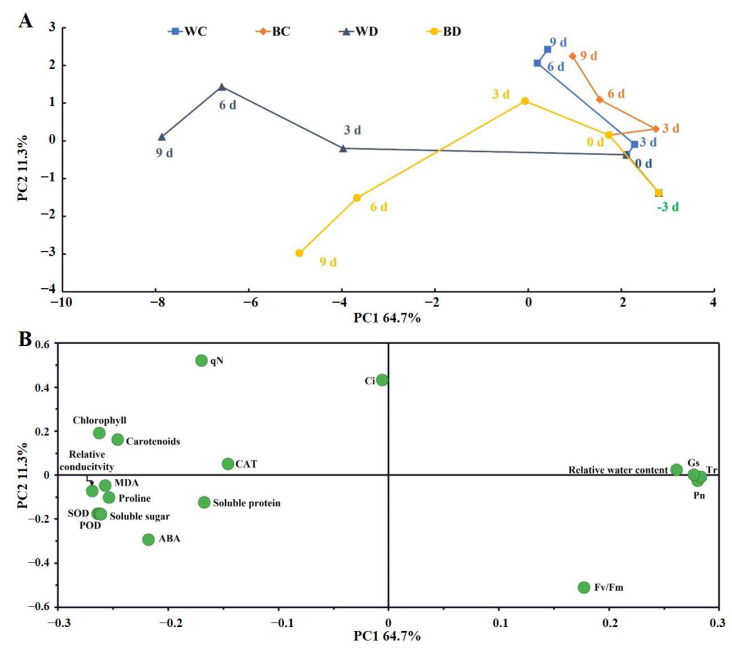
Time-related trajectory plot (**A**) and principal components analysis (PCA) plot (**B**) showing the time-related responses of growth status in potato leaves treated with drought or/and EBR pretreatment.

There were clear differences in the growth status of potato leaves at different times during the experimental period and among different treatments, and greater changes in the growth status of plants were associated with greater distances from −3 d and WC (Figure 5A). Changes in distances from 0 d were large for both the WD and the BD plants. The largest change in the first 3 d was observed in the WD plants, and the largest change from 3 to 6 d was observed in the BD plants. Changes were less pronounced in the EBR-treated plants than in the WD plants throughout the drought period. The distance from the origin was shorter for the EBR-treated plants at 6 d than for the WD plants at 3 d, and this distance in the EBR-treated plants at 6 d was approximately half of that in the WD plants at 9 d. These results indicated that the effects of drought stress on potato plants were delayed by the EBR pretreatment. The factors that loaded most strongly on the principal components were the photosynthetic parameters (Tr, Gs, and *Pn*) and antioxidant enzymes (SOD and POD); the main contributing factors that distinguished the WD plants from the BD plants were qN in WD and ABA in BD (Figure 5B).

### 3.6. RNA-Seq and Differential Gene Expression

Twelve cDNA libraries from four treatments were sequenced, and a total of 508,716,686 raw reads were generated. High-quality clean reads accounted for 97.55% of the total reads, and the average Q30 (0.1% error rate) was 92.89%, suggesting that the clean reads sequenced via RNA-seq were high quality and that there was a low mismatch rate. These reads were, thus, mapped to the potato reference genome (Table S3).

**Figure 6:**
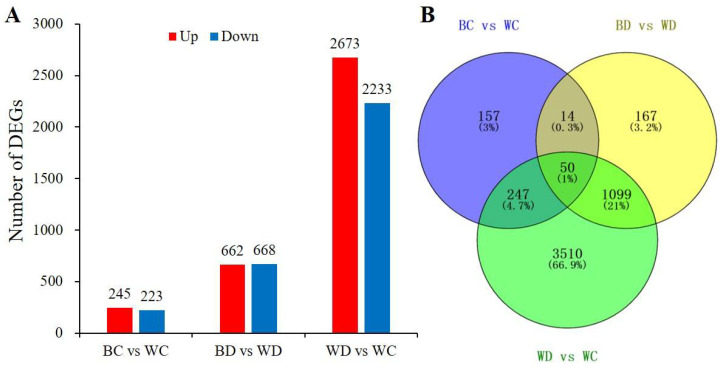
The differentially expressed genes (DEGs) in potato leaves treated with drought or/and EBR pretreatment. (**A**) The number of upregulated and downregulated DEGs. (**B**) Venn diagram of DEGs among the comparison groups.

The number of differentially expressed genes (DEGs) varied from 468 (BC vs. WC) to 4906 (WD vs. WC) for the three different comparisons (Figure 6A, Table S4). The number of upregulated and downregulated DEGs was 662 and 668 for BD vs. WD, respectively. Only 157 DEGs were exclusively present in BC vs. WC, while 3510 DEGs were exclusively identified in the WD vs. WC comparison. A significant proportion of the DEGs (1099 genes, accounting for 21%) were commonly regulated in the BD vs. WD and WD vs. WC comparisons. A total of 50 DEGs were commonly regulated in the three comparisons (Figure 6B).

### 3.7. Verification of RNA-Seq Data

A total of 22 DEGs were randomly selected for quantitative real-time PCR (qRT-PCR) detection. The expression patterns of nearly all these genes were similar according to the two methods, suggesting the data obtained from the RNA-seq analysis were robust (Figure S2).

### 3.8. GO and KEGG Enrichment of DEGs

**Figure 7:**
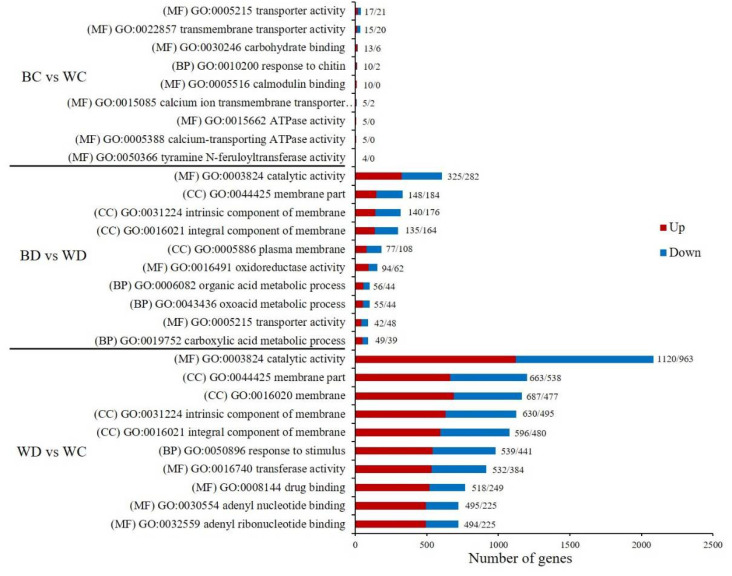
Gene ontology (GO) enrichment analysis of DEGs among comparison groups. Top 9 or 10 enriched terms with *p*-values < 0.05 in each comparison are shown. The upregulated and downregulated genes of the enriched terms are shown in red and blue bars, respectively, and the number of genes is shown in the bar graph. MF, molecular function; BP, biological process; CC, cellular component.

A gene ontology (GO) analysis was conducted to obtain functional annotations of DEGs for each comparison (Table S5). The top 10 enriched terms belonged to the three main GO categories: molecular function (MF), cellular component (CC), and biological process (BP) (Figure 7). There were four highly enriched terms for the BD vs. WD and the WD vs. WC comparisons: catalytic activity (GO:0003824) in MF, membrane part (GO:0044425), intrinsic component of the membrane (GO:0031224), and integral component of the membrane (GO:0016021) terms in CC. In addition, transporter activity (GO:0005215) in the MF was highly enriched in both the BD vs. WD and the BC vs. WC comparisons.

KEGG (Kyoto encyclopedia of genes and genomes) enrichment pathway analysis revealed several pathways involved in the EBR and the drought stress response (Table 1, Table S6). Plant hormone signal transduction had the highest number of DEGs in the WD vs. WC, the BD vs. WD, and the BC vs. WC comparisons (63, 20 and 7, respectively), and 75% (15/20) of the DEGs were significantly upregulated by exogenous application of EBR under drought (BD vs. WD). Starch and sucrose metabolism, alpha-linolenic acid metabolism, flavonoid biosynthesis, and carotenoid biosynthesis were significantly enriched in both the BD vs. WD and the WD vs. WC comparisons, and MAPK signaling pathways were significantly enriched in both the WD vs. WC and the BC vs. WC comparisons. Furthermore, circadian rhythm was significantly enriched in the BD vs. WD comparison, and photosynthesis (including antenna proteins and carbon fixation) and galactose metabolism were significantly enriched in the WD vs. WC comparison.

### 3.9. Drought-Responsive Genes under EBR Applications

**Figure 8:**
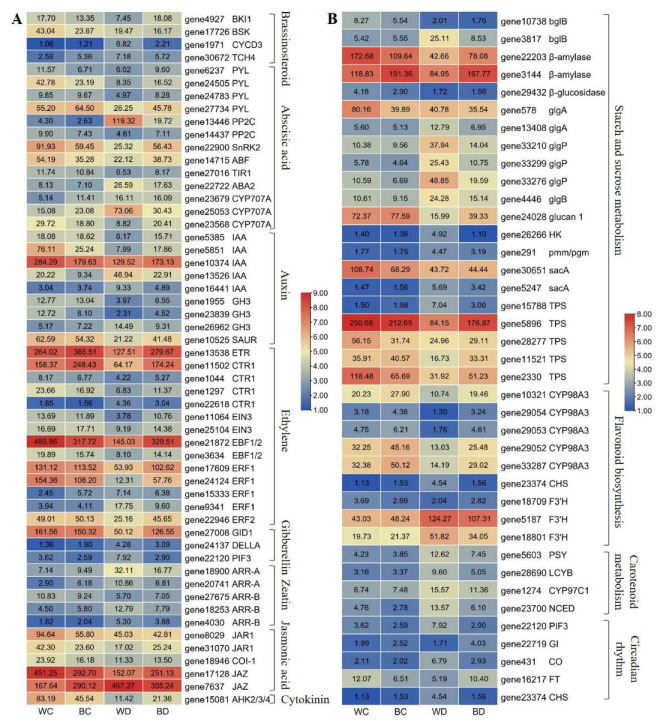
Heatmap of DEGs in KEGG pathway enrichment. (**A**) Plant hormone signal transduction pathways. (**B**) Starch and sucrose metabolism, flavonoid biosynthesis, carotenoid metabolism, and circadian rhythm related pathways.

Several DEGs related to hormone signal transduction were involved in EBR and the drought response, including BR, ABA, auxin, ethylene, gibberellin, zeatin, jasmonic acid, and cytokinin (Figure 8A). Under drought stress, the expression levels of BRI1 kinase inhibitor *BRI1 KINASE INHIBITOR1 (BKI1)* (gene4927), ABA receptor *PYR1-LIKE PROTEINs* (*PYL*) (gene24505) and *SUCROSE NON-FERMENTING 1 RELATED PROTEIN KINASE 2s* (*SnRK2*) (gene22900), auxin-responsive protein *IAA* (gene5385, gene5851) and *SAUR* (gene10525), ethylene receptor *ETR* (gene13538), serine/threonine-protein kinase *CTR1* (gene11502), ethylene-insensitive protein *EIN3* (gene11064), EIN3-binding F-box protein *EBF1/2* (gene21872), ethylene-responsive transcription factor *ERF1* (gene17609, gene24124), gibberellin receptor *GID1* (gene27008), and cytokinin receptor *AHK2/3/4* (gene15081) were significantly downregulated. However, exogenous application of EBR affected the regulation of these hormone-related genes by drought stress and induced their expression. Furthermore, the expression of cyclin D-type protein *CYCD3* (gene1971), abscisic acid 8′-hydroxylase (*CYP707A*) (gene25053), ABA signaling negative regulators serine/threonine protein phosphatase 2C (*PP2C*) (gene13446), and phytochrome-interacting factor *PIF3* (gene22120) was upregulated by drought; however, their expression was stabilized to the level observed in the WC plants after the exogenous application of EBR.

In the starch and sucrose metabolic pathways, drought stress significantly upregulated the expression levels of beta-glucosidase (*bglB*) (gene3817), starch phosphorylase (*glgP*) (gene33210, gene33276), and hexokinase (*HK*) (gene26266), and downregulated the expression levels of beta-amylase (gene3144), trehalose 6-phosphate synthase/phosphatase (*TPS*) (gene5896, gene11521) and 1,3-beta-glucosidase (*glucan 1*) (gene24028); the exogenous application of EBR resulted in the opposite expression patterns. In the flavonoid biosynthetic pathway, the expression of chalcone synthase (*CHS*) (gene23374) and flavonoid 3′-monooxygenase (*F3′H*) (gene5187, gene18801) was significantly upregulated, and the expression of coumaroylquinate (coumaroylshikimate) 3’-monooxygenase (*CYP98A3*) (gene29052, gene29054, gene33287) was inhibited by drought stress; however, the exogenous application of EBR downregulated the expression of these genes induced by drought stress. The expression of multiple carotenoid metabolic genes, such as phytoene synthase (*PSY*) (gene5603), lycopene beta-cyclase (*LCYB*) (gene28690), carotene epsilon-monooxygenase (*CYP97C1*) (gene1274), and 9-cis-epoxycarotenoid dioxygenase (*NCED*) (gene23700) was upregulated under drought stress, and the exogenous application of EBR downregulated the expression of these genes under drought stress. The downregulation of *FLOWERING LOCUS T* (*FT*) (gene16217) and the upregulation of zinc finger protein *CONSTANS* (*CO*) (gene431) under drought stress were reversed after the exogenous application of EBR (Figure 8B).

In addition to the drought-responsive genes in the above pathways, exogenous EBR application had an effect on the regulation of the photosynthesis and MAPK signaling pathways under drought. Drought significantly upregulated the expression of more than 80% (36/44) of the DEGs involved in the photosynthesis pathway, including 12 antenna proteins, 10 carbon fixation proteins, and 14 photosynthesis-related proteins, especially light-harvesting complex I/II chlorophyll a/b binding protein (*LHCA/B*), ribulose-bisphosphate carboxylase (*rbcS*), fructose-bisphosphate aldolase (*ALDO*), transketolase (*tktA/B*), ferredoxin (*petF*), cytochrome c6 (*petJ*), *psaK* and *psaO* of photosystem I, and *psb27/28* and *psbR* of photosystem II. However, the expression of *LHCB1* (gene29953), *LHCB3* (gene23868), and *petJ* (gene26569) was significantly downregulated after the exogenous application of EBR in the BD plants relative to that in the WD plants. Drought significantly downregulated the expression of more than 65% (21/32) of the DEGs involved in the MAPK signaling pathway, such as *ETR*, *CTR1*, *EIN3*, *EBF1/2*, *ERF1*, *PYL*, and *SnRK2*, and the exogenous application of EBR mitigated the downregulation of these genes under drought stress (Figure S3).

### 3.10. Expression Patterns of Genes Involved in BR and ABA Biosynthesis

**Figure 9:**
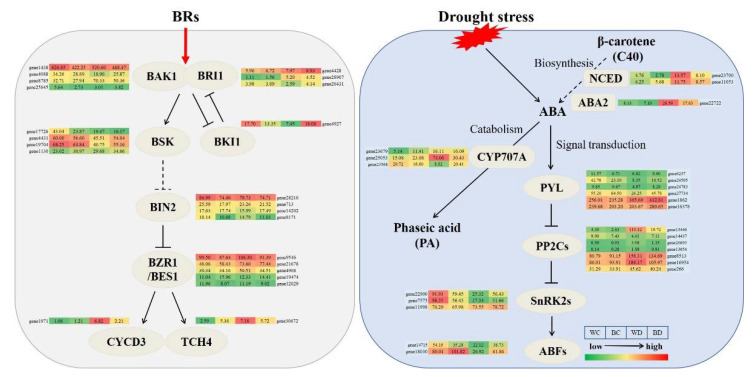
Molecular regulation mechanism of exogenous EBR improving potato drought resistance in BR signaling pathway and ABA metabolic pathway. Red and green denoted high and low relative expression, respectively.

To explore the effect of exogenous EBR on the core hormonal pathways of potatoes under drought stress, the expression patterns of major genes involved in BR signaling and ABA metabolic pathways were investigated (Figure 9). The expression level of *BKI1* was upregulated in the BD plants compared with the WD plants, and there were no differences in the expression of *BRI1-ASSOCIATED KINASE1 (BAK1)*, *BR INSENSITIVE1 (BRI1)*, *BR SIGNALING KINASES (BSK)*, *BIN2,* and *BRASSINAZOLE-RESISTANT1/BRASSINOSTERIOD INSENSI-TIVE1-EMS-SUPPRESSOR1 (BZR1/BES1)* between the BD and the WD plants. The expression of both *CYCD3* and xyloglucosyl transferase *TCH4* was upregulated by drought stress, and partially downregulated after exogenous EBR application. The expression of *PYL*, *SnRK2s*, and *ABA-RESPONSIVE ELEMENT-BINDING FACTORs* (*ABFs*) was upregulated in the BD plants compared with the WD plants, and the expression of *NCED*, *ABA2*, *PP2Cs*, and *CYP707A* were downregulated in the BD plants compared with the WD plants.

## 4. Discussion

**Figure 10:**
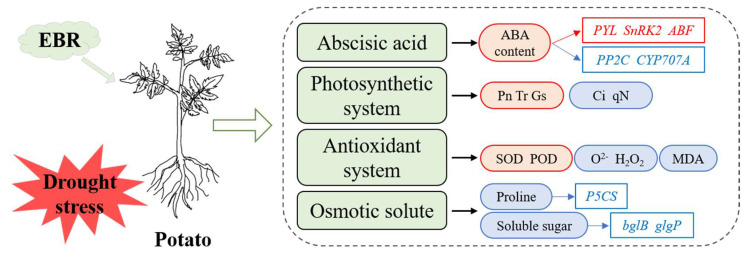
Physiological and molecular regulatory mechanisms by which exogenous EBR application enhances the drought resistance of potatoes. Red and blue boxes indicate the increases and decreases in the content of substances and gene expression levels, respectively.

Drought is an important factor limiting the yield and quality of potatoes. Plants have evolved complex regulatory mechanisms to adapt to harsh environmental conditions, such as droughts [50]. BRs are important plant phytohormones that play a key role in plant growth and stress responses. In this study, the physiological and molecular mechanisms underlying the alleviation of drought stress by EBR pretreatment were studied in potatoes. EBR pretreatment facilitated adaptation to drought stress in potatoes by regulating physiological responses, including the ABA content, photosynthetic characteristics, antioxidant systems, and osmotic solutes. Transcriptome data were used to identify drought genes in response to EBR application, and the molecular mechanisms by which this BR alleviates drought stress in potatoes were characterized (Figure 10). This is the first study, to our knowledge, to elucidate the mechanisms by which a BR alleviates drought stress in potatoes.

### 4.1. Response of Hormone Signals under Drought Stress after EBR Pretreatment

The regulation of plant drought tolerance involves key signaling metabolites and plant hormones, as well as their activity-regulating proteins [51]. In this study, the expression of the BR biosynthetic genes *CYCD3* and *TCH4* was upregulated under drought stress, and EBR pretreatment stabilized their expression levels. This might be explained by the alleviation of the inhibition of the BR-related kinase inhibitor *BKI1* on the BR signaling pathway due to the downregulation of *BKI1* under drought [52], which eventually led to the upregulation of BR biosynthetic genes. BRs and ABA are maintained in a dynamic balance to mediate plant growth and stress responses through signal communication at the transcriptional level and to regulate the protein post-translational modification levels, and the complex regulatory networks among BRs, ABA, and other hormone signals [29,30,53]. In this study, the expression level of the ABA biosynthetic gene *NCED* was upregulated, which promoted the accumulation of ABA, and a high ABA content was maintained following EBR pretreatment by inhibiting the transcription of the ABA catabolic gene *CYP707A*. Furthermore, EBR pretreatment regulated the ABA signal pathway through the inhibition of the phosphatase activity of *PP2Cs* proteins by upregulating the expression of its receptor *PYL* [51], which eventually increased the expression of *SnRK2s* and *ABFs*, indicating that *PYL* is a positive regulator underlying the alleviation of drought stress in potatoes by EBR, that revealed the important role of the ABA-dependent signaling pathway in EBR alleviating drought stress in potatoes. In addition, EBR pretreatment increased the expression levels of the ethylene receptor and transcription factors *ETR*, *CTR1*, *EBF1/2*, *ERF1*, and the gibberellin receptor *GID1* under drought, suggesting that these hormone genes might positively regulate the response to drought stress following EBR treatment in potatoes.

### 4.2. Effect of EBR on Growth and the Photosynthesis of Potato under Drought Stress

Cellular water loss is the first major consequence of drought stress [4]. In most instances, drought stress causes plant stomatal closure and a reduction in the rate of photosynthesis, which results in phenotypes, such as wilting and leaf rolling [54,55]. In this study, EBR pretreatment alleviated the decrease in the water content, and the potato phenotype was closer to that of its normal growth state, which is consistent with the observations in *Robinia pseudoacacia* and tomatoes [23,56]. The reductions in *Pn*, Gs, and Ci at 3 d of drought stress were directly related to stomatal closure in potatoes [57]. To compensate for the damage to the photosystems induced by drought stress, the expression of most photosynthesis-related genes, such as *LHCA/B*, *rbcS*, *psaK*, and *psaO* of photosystem I, and *psb27/28* and *psbR* of photosystem II, was upregulated. In the later stages of the drought period (6 d and 9 d), the reduction in the rate of photosynthesis was mainly caused by nonstomatal limiting factors in mesophyll cells, such as the reduction in the activity of Calvin cycle-related enzymes [58]. The EBR pretreatment maintained the normal status of the stomata and the activity of assimilation enzymes, which resulted in the maintenance of a higher rate of photosynthesis. The level of qN was used to quantify photoprotective and photoinhibitory processes [59]. Our results indicate that EBR pretreatment reduces the residual excitation energy that cannot be used for photosynthetic electron transfer and heat dissipation, which protects the photosynthetic system. The chlorophyll content remained high under drought stress, and this finding is consistent with the results of previous studies. Plants displaying these features possess a “non-functional stay-green” phenotype, and increases in chlorophyll concentration are caused by factors, such as leaf growth restriction and cell water loss [60].

### 4.3. Effect of EBR on the ROS Scavenging System of Potato under Drought Stress

Under drought stress, ROS, such as O^2−^ and H_2_O_2_, resulted in membrane lipid peroxidation and a simultaneous increase in the MDA content; however, these effects were ameliorated with the EBR pretreatment, which was indicated by the delay in the peak of antioxidant enzymes (SOD, POD, and CAT) activity in potatoes. Protection from these effects (at least in part) was also provided in plants, such as *Robinia pseudoacacia* [56] and tomatoes [23] under EBR pretreatment, because of increased antioxidant enzyme activities. EBR pretreatment may also initiate a nonenzymatic system that regulates the expression of glutathione (GSH) and flavonoid genes, and quenches ROS in potatoes. Consistent with this possibility, some of the upregulated DEGs included genes encoding glutathione s-transferase (*GST*) and flavonoid synthase (*CYP98A3*).

### 4.4. Effect of EBR on the Osmotic Solutes of Potato under Drought Stress

Osmoregulatory substances play important roles in mediating tolerance to drought stress [23]. In this study, the content of soluble sugar increased more slowly in BD plants than in WD plants, and the accumulation of soluble sugar was mainly derived from the decomposition of macromolecules, such as starch and the photosynthetic product sucrose, in a drought [55]. Consistent with this observation, drought stress upregulated the expression of the starch phosphorylase genes *bglB* and *glgP*. In addition, the expression of the proline biosynthesis-related enzyme delta-1-pyrroline-5-carboxylate synthetase (*P5CS*) was upregulated by drought, which resulted in the accumulation of proline under drought stress in potatoes.

## 5. Conclusions

The mechanism by which exogenous EBR enhances the drought resistance of potatoes was revealed by physiological and comparative transcriptome analysis. Exogenous EBR increased the ABA content, photosynthetic capacity, and the activities of antioxidant enzymes, and decreased the content of ROS, MDA, proline, and soluble sugar in potatoes under drought stress. The DEGs associated with the response to EBR under drought stress were detected, and the regulatory genes associated with BR signaling and biosynthesis, as well as ABA metabolic pathways, were identified. These findings enhance our contribution to the understanding of the mechanism by which exogenous EBR enhances the drought resistance of potatoes, and the drought-responsive genes identified following the application of EBR provide targets for future molecular breeding.

## Figures and Tables

**Table 1:** Kyoto encyclopedia of genes and genomes (KEGG) enrichment analysis of DEGs among comparison groups.

Comparison	KEGG Pathway	Upregulated DEGs Number	Downregulated DEGs Number	*p*-Value
BC vs. WC	MAPK signaling pathway-plant	3	2	0.0349
	Plant-pathogen interaction	5	1	0.0444
	Plant hormone signal transduction	2	5	0.0494
BD vs. WD	Circadian rhythm-plant	3	4	0.0010
	Starch and sucrose metabolism	4	6	0.0013
	Alpha-linolenic acid metabolism	7	0	0.0045
	Plant hormone signal transduction	15	5	0.0057
	Flavonoid biosynthesis	4	2	0.0111
	Carotenoid biosynthesis	2	2	0.0387
WD vs. WC	Photosynthesis-antenna proteins	12	0	0.0000
	Starch and sucrose metabolism	16	12	0.0000
	Carbon fixation in photosynthetic organisms	10	6	0.0005
	Photosynthesis	14	2	0.0021
	Plant hormone signal transduction	24	39	0.0016
	Alpha-linolenic acid metabolism	3	13	0.0074
	Flavonoid biosynthesis	6	9	0.0069
	Galactose metabolism	7	7	0.0063
	Carotenoid biosynthesis	7	3	0.0290
	MAPK signaling pathway-plant	11	21	0.0497

## Data Availability

All data supporting the findings of this study are available within the paper and within its Supplementary Materials published online.

## Supplementary Materials

The following supporting information can be downloaded at: https://www.mdpi.com/article/10.3390/antiox11091701/s1, Figure S1: plant phenotype of potatoes after 9 days of drought with different concentrations of EBR pretreatment; Figure S2: expression pattern of 22 selected DEGs obtained by qRT-PCR and RNA-seq; Figure S3: heatmap of DEGs enriched in photosynthesis and MAPK signaling pathways; Table S1: primer sequences used in this study for qRT-PCR; Table S2: physiological parameters in potato leaves with drought treatment and EBR pretreatment; Table S3: sequencing statistics of the transcriptome from four treatments of potato leaves; Table S4: a dataset of significantly up- and down-regulated DEGs; Table S5: GO enrichment analysis of DEGs; Table S6: KEGG enrichment analysis of DEGs.
