# Supplementary figures and images for: Physiological and Comparative Transcriptome Analysis Reveals the Mechanism by Which Exogenous 24-Epibrassinolide Application Enhances Drought Resistance in Potato (Solanum tuberosum L.)
Source: Antioxidants (Basel). 2022 Aug 30;11(9):1701. doi: 10.3390/antiox11091701 (PMC9495798; doi:10.3390/antiox11091701)

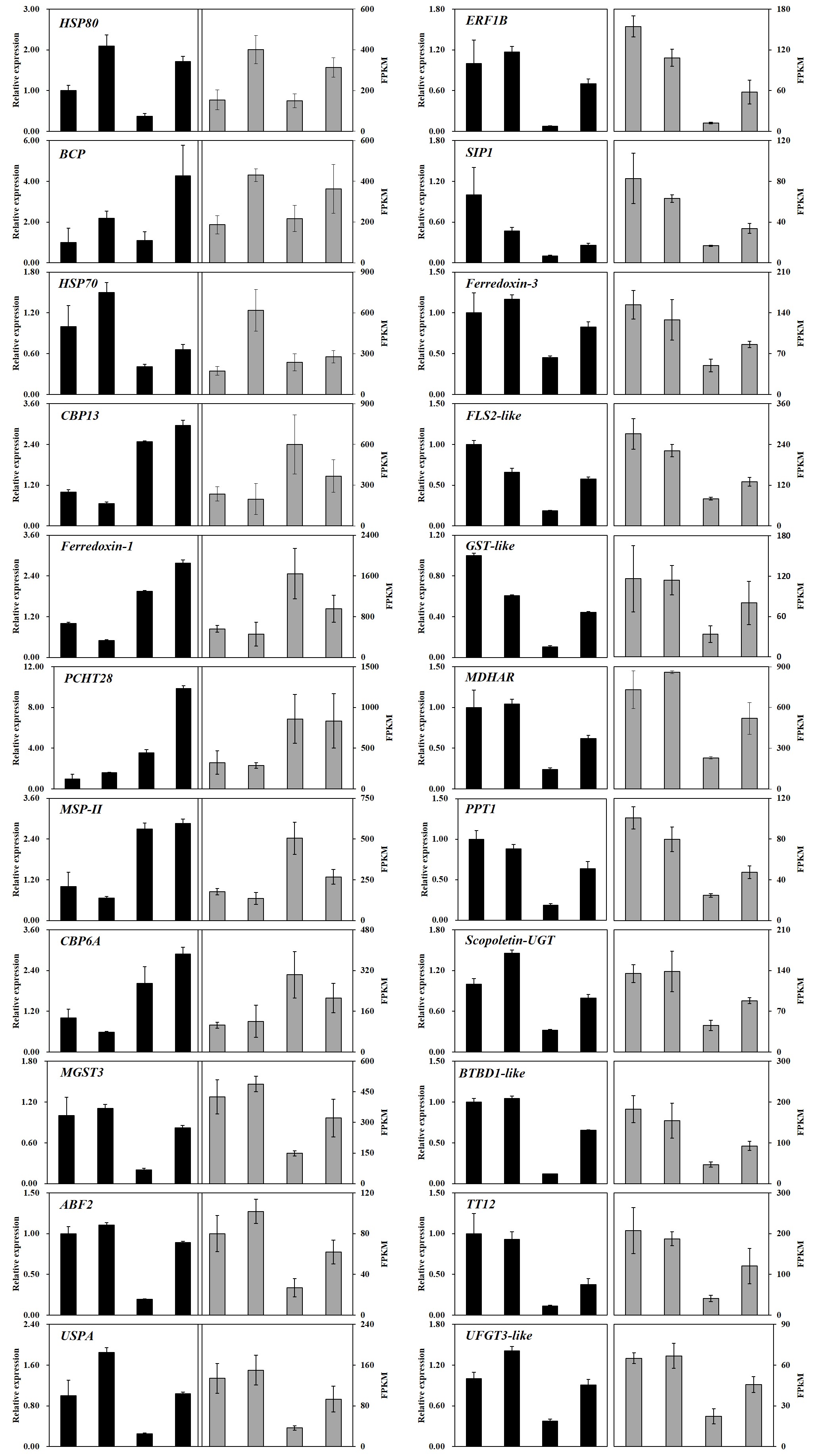

Supplement: Supplementary file 1 [file antioxidants-11-01701-s001.zip › Supplementary/Figure S1 Expression pattern of 22 selected DEGs obtained by qRT-PCR and RNA-seq.jpg]

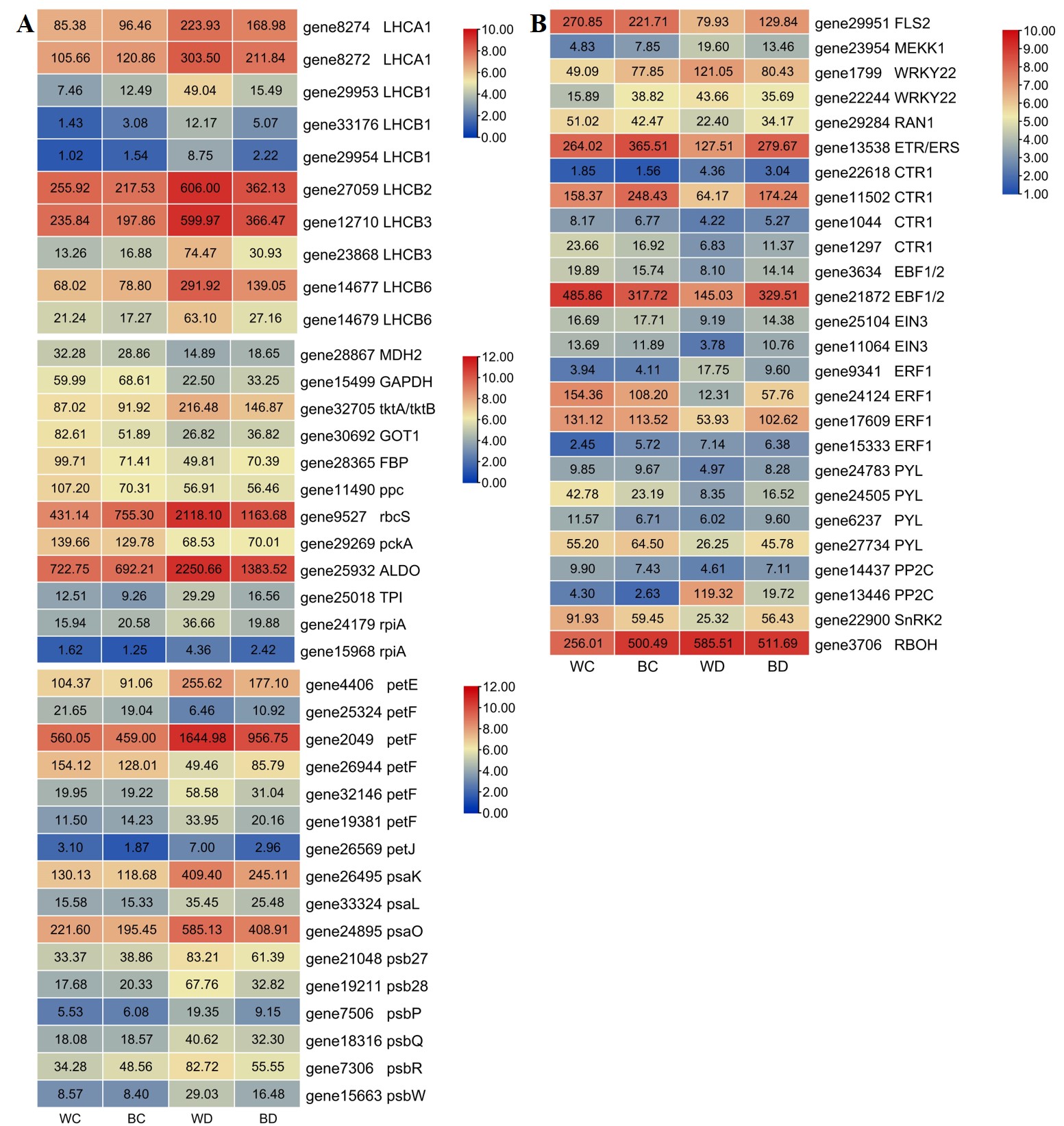

Supplement: Supplementary file 1 [file antioxidants-11-01701-s001.zip › Supplementary/Figure S2 Heatmap of DEGs enriched in photosynthesis and MAPK signaling pathways.jpg]

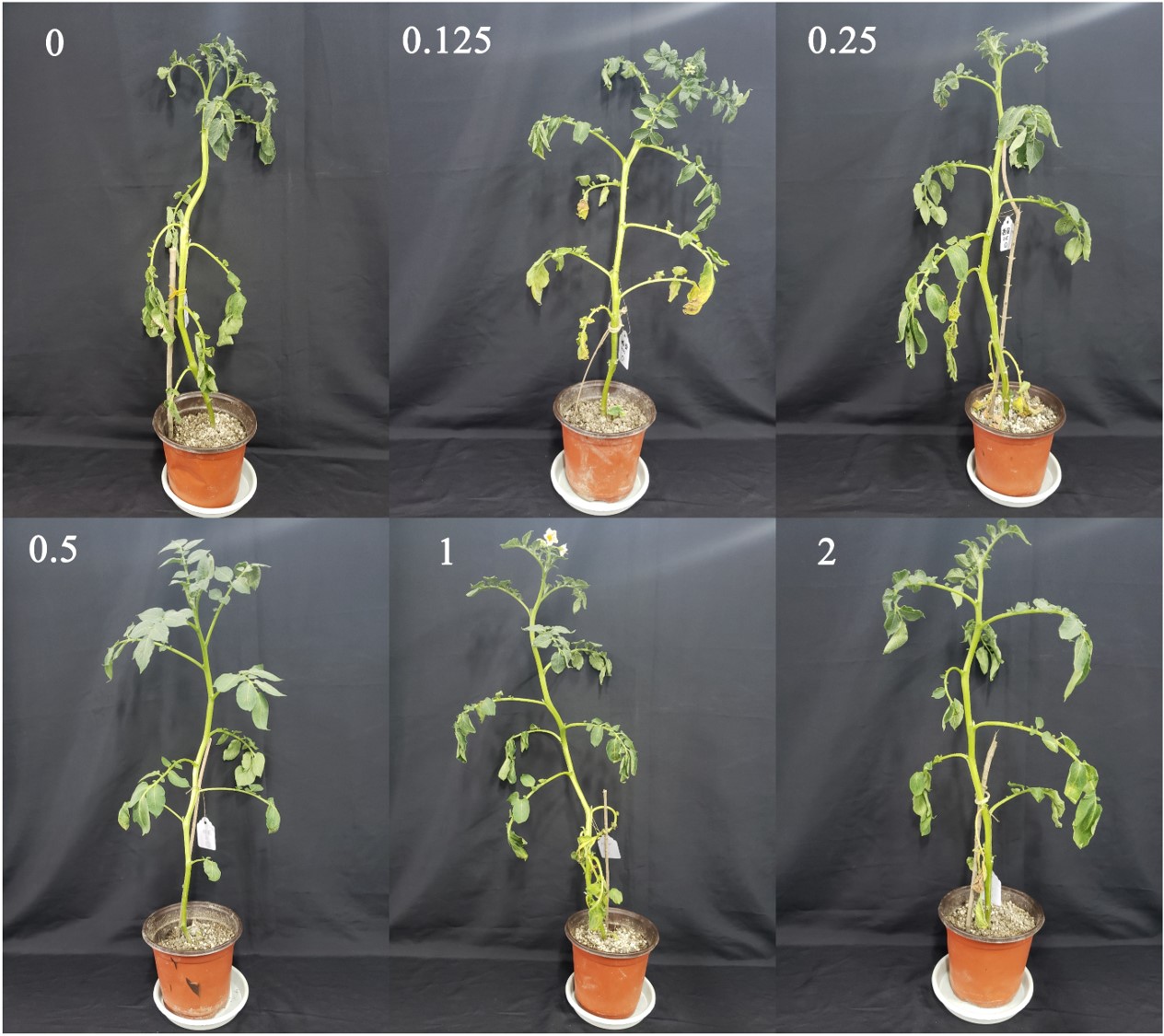

Supplement: Supplementary file 1 [file antioxidants-11-01701-s001.zip › Supplementary/Figure S3 Plant phenotype of potatoes after 9 days of drought with different concentrations of EBR pretreatment.jpg]
